# Accuracy of Digital Photography and Intraoral Scanning for Tooth Shade Selection in Digital Prosthodontics: A Comparative Clinical Study Using Spectrophotometry as Reference

**DOI:** 10.3390/dj14050308

**Published:** 2026-05-18

**Authors:** Luciana Maria Goguta, Mirela Frandes, Melania Teodora Nāşcuţiu, Alina Cudera, Anca Jivanescu

**Affiliations:** 1 Department of Prosthodontics, Faculty of Dentistry, “Victor Babes” University of Medicine and Pharmacy, 300041 Timisoara, Romania; goguta.luciana@umft.ro (L.M.G.); jivanescu.anca@umft.ro (A.J.); 2 Digital and Advanced Technique for Endodontic, Restorative, and Prosthetic Treatment (TADERP) Research Center, “Victor Babes” University of Medicine and Pharmacy, 300041 Timisoara, Romania; 3 Department of Functional Sciences-Medical Informatics and Biostatistics, “Victor Babes” University of Medicine and Pharmacy, 300041 Timisoara, Romania; 4 Center for Modeling Biological Systems and Data Analysis, “Victor Babes” University of Medicine and Pharmacy, 300041 Timisoara, Romania

**Keywords:** tooth shade selection, digital prosthodontics, intraoral scanner, digital photography, spectrophotometry, CIELAB, ΔE, Bland–Altman analysis

## Abstract

**Background**: The integration of digital technologies into prosthodontics has significantly improved clinical workflows, from intraoral data acquisition to restoration design and fabrication. Accurate tooth shade selection remains a critical step in achieving optimal aesthetic outcomes, with spectrophotometry considered the gold standard. However, alternative digital methods such as intraoral scanners and digital photography are increasingly used in clinical practice. **Objective**: This study aimed to evaluate the accuracy of intraoral scanning and standardized digital photography for tooth shade selection within a digital prosthodontic workflow, using spectrophotometric measurements as reference. **Methods**: Twenty participants were included in this clinical study. Tooth color was recorded using a spectrophotometer (reference method), an intraoral scanner (Medit i700), and standardized digital photography, which was analyzed with a Digital Color Meter. Color differences (ΔE) were calculated using the CIELAB formula. Statistical analysis included one-sample and independent-samples *t*-tests, as well as Bland–Altman analysis to assess agreement between methods. **Results**: Digital photography showed significantly higher ΔE values (19.37 ± 2.45) compared to the clinical acceptability threshold (ΔE = 3.3; *p* < 0.001), indicating poor accuracy. The intraoral scanner demonstrated lower ΔE values (5.53 ± 0.62; *p* < 0.001) but still exceeded the acceptable limit. Bland–Altman analysis confirmed poor agreement for digital photography and a reduced, though still suboptimal, agreement for the intraoral scanner relative to spectrophotometry. Nonetheless, the intraoral scanner demonstrated significantly better agreement than digital photography, supporting its preferential use in digital prosthodontic workflows. **Conclusions**: Within the context of digital prosthodontics, digital photography cannot be considered a reliable method for shade matching. Intraoral scanners provide improved consistency but do not achieve clinically acceptable accuracy compared to spectrophotometry. Spectrophotometry remains the gold standard, while intraoral scanning may serve as a complementary tool in digital workflows. Future studies with larger samples are needed to confirm these findings.

## 1. Introduction

Digital shade selection represents an essential component of the modern digital prosthodontic workflow, complementing intraoral scanning and CAD/CAM-based restoration design. One of the key factors for success in aesthetic restorative dentistry is achieving harmony between the color of the restoration and that of the remaining teeth or existing restorations [1]. Color selection in dentistry is a complex process that demands technical knowledge, clinical experience, and a precise protocol to achieve satisfactory results. Color is a complex and subjective psychophysical phenomenon. It can be perceived in different ways and depends directly on the object, the light source, and the observer [2]. Therefore, color matching in dentistry is quite complex because many factors influence it. The main challenges include subjective differences among evaluators, the multicolored nature of teeth, and the limitations of dental shade guides, which do not fully capture the natural tooth color range [3]. Additionally, as materials and technologies have improved and patient expectations have increased, matching tooth color has become more difficult. To ensure harmony between the restoration’s color and that of the surrounding teeth or existing restorations, it is crucial to accurately determine their shade and communicate the details clearly and precisely to the laboratory.

In a dental office, tooth color can be selected using various techniques, including traditional methods such as shade guides and digital methods. Digital techniques for assessing, communicating, and reproducing color in dentistry have been created to improve the accuracy of color matching. These methods reduce dependence on subjective observer factors and provide more reproducible measurements.

In the traditional method, color selection is a process that requires careful attention and more time. The Vitapan 3D Master shade guide (VITA Zahnfabrik, Bad Säckingen, Germany) helps practitioners quickly and accurately choose a shade. It considers three color dimensions: brightness, saturation level, and hue. It is organized into five brightness levels, three intensity levels for the medium shade (M), and two intensity levels for yellowish (L) and reddish (R) shades [4]. The traditional method of selecting colors in dentistry, which relies on shade guides, can be prone to errors due to factors such as subjectivity, eye fatigue, and external influences. The inherent subjectivity of the shade-matching process is what efforts aim to address [5]. However, studies have shown that up to 80% of patients are dissatisfied with noticeable differences in shade [6]. Shade matching is usually straightforward with uniformly colored objects; however, teeth vary in color and transparency, making them harder to match [7]. Lighting also plays a crucial role in color perception and greatly impacts the accuracy of shade matching. Therefore, it is often recommended to use controlled and standardized lighting when evaluating dental shades, both by observers and by instruments [8]. It is also known that the ability to choose a shade varies among individuals and even for the same person at different viewing times [9]. Conversely, chromatic anomalies influence accurate shade selection, and these deficiencies are more common among men. About 8% of men and 0.5% of women have some level of color vision deficiency. This is one reason why women are considered more capable of matching shades than men [6]. Another factor to consider in shade matching is the observer’s clinical experience, which can affect their ability to match [10].

The digital devices used to determine color in dental prosthetics include digital cameras, spectrophotometers, colorimeters, computerized colorimetric analysis of digital images, and hybrid devices [4]. The primary benefit of dental shade-matching instruments is their ability to reduce imperfections and inconsistencies associated with visual shade matching [10]. Studies indicate that spectrophotometers and colorimeters deliver more consistent results in shade matching [8]. In dentistry, the spectrophotometer is regarded as the most precise instrument for matching the color of restorations to natural teeth due to its practicality and effectiveness [9].

In the dental office, one of the spectrophotometers used is the Vita EasyShade^®^ V (VITA Zahnfabrik, Bad Säckingen, Germany). The Vita EasyShade^®^ V spectrophotometer measures the spectral distribution of light and converts it into a recognized international or numeric color value. It provides valuable support to dental practices by accurately determining the color of teeth or restorations, aiding in choosing the best restorative materials. It is also compact and ergonomic, with a touchscreen display. The Vita EasyShade^®^ V measures the natural tooth color and displays values according to the Vita 3DMaster and Vita Classic shade guides, as well as L*a*b* and L*C*h° values [10]. Colorimeters are designed to measure color as perceived by the human eye. They filter light into four regions of the visible spectrum to determine an object’s color, but they are less accurate than spectrophotometers [4].

Digital photography is an essential alternative to color measurement tools and enables a more effortless transfer of data from the oral cavity to the technician [11]. However, it has been noted that interpreting tooth color with digital photography relies heavily on the individual and can be subjective and often inaccurate [12]. Current intraoral scanners also have color determination functions. Devices such as TRIOS 3 (3Shape Trios A/S, Copenhagen, Denmark) allow for color determination from any point, according to both the Vita System 3D-Master scale and the Vita Classical A1–D4 scale. Recent research indicates that intraoral scanners are reliable devices for selecting colors, but their accuracy is not optimal, and selection must be combined with other known methods [13,14,15,16,17,18]. In practice, intraoral scanners are not yet widely used as a primary method for color matching in clinical settings, and their use for shade determination is not yet standardized in the medical literature [13,14].

Human eyes are highly skilled at detecting small color differences between natural teeth and prosthetics. Instrumental color analysis, combined with advanced manufacturing techniques, offers an effective way to minimize these differences during the manufacturing process [18]. Most digital systems utilize the ΔE parameter, part of the CIELAB system, to measure the color difference between a selected restoration and the tooth being restored. ΔE quantifies the difference between two colors, and the aim is to get the smallest possible value, indicating a precise color match. ΔE is calculated from the parameters of the CIELAB color space: L*, a*, and b*. The CIELAB system includes these indices: L*, for lightness; a*, which corresponds to the red–green axis; and b*, representing the yellow–blue axis [4].

The primary hypothesis of this study was that the intraoral scanner (Medit i700) would provide more accurate shade selection than standardized digital photography, as measured by spectrophotometric measurements. The second hypothesis proposed that intraoral scanning would achieve clinically acceptable color accuracy (ΔE ≤ 3.3) for shade matching. The third hypothesis was that standardized digital photography, when analyzed with a calibrated color measurement tool, would yield reliable shade-matching results comparable to those of spectrophotometry.

## 2. Materials and Methods

### 2.1. Study Design and Participants

The study was carried out at the Clinic of Prosthodontics, Research Center TADERP, Faculty of Dental Medicine, University of Medicine and Pharmacy “Victor Babes”, Timisoara, Romania. Twenty participants, including prosthodontic residents and young assistant professors aged 27–35 years, were involved in the study. The study received approval from the University’s Ethical Committee (No. 3/31 January 2023), in accordance with the Declaration of Helsinki.

The inclusion criteria were as follows:

- The absence of anterior tooth loss;
- The absence of anterior teeth discoloration caused by direct/indirect restorations, root canal treatments, or other factors.

Exclusion criteria include the following:

- Participants who had undergone whitening treatments in the last 6 months;
- Poor oral hygiene.

All participants received standardized written information explaining the purpose of the study and shade measurement methods before any clinical procedure. Both subgroups (prosthodontic residents and young assistant professors) received identical information, ensuring equivalent knowledge of the study protocol regardless of professional status. Before starting the clinical procedures, they were asked to brush their teeth for approximately 2 min using a standardized oral hygiene protocol and to avoid makeup products, such as lipstick, that could influence color perception. No formal professional prophylaxis was performed, as good oral hygiene was an implicit inclusion criterion.

The study population consisted of a convenience sample of staff and trainees recruited from our institution during the study period. Although no formal a priori power calculation was performed, this sample size is comparable to those reported in similar preliminary clinical investigations in the dental shade-matching literature [16].

### 2.2. Color Measurement Procedures

Initially, color measurements were taken using a spectrophotometer, the Vita EasyShade^®^ V (Vita Zahnfabrik, Bad Säckingen, Germany), which is considered the gold standard. The reference point for these measurements was the center of the right maxillary central incisor. Later, the Medit i700 intraoral scanner (Medit Corp., Seoul, Republic of Korea) was used to scan the right maxillary central incisor according to the manufacturer’s instructions. After completing the digital record and documenting personal details, the diagnostic scan icon was selected. The operator can display the 3D-Master tooth shades measured in the middle third of the vestibular surface of the tooth by placing the cursor over the tooth after selecting the tooth shade icon.

Since the Medit i700 intraoral scanner provides shade codes in the Vita 3D-Master notation system (e.g., 1M1, 2M1) rather than direct L*, a*, b* values, these codes were subsequently converted to L*, a*, and b* values using the standardized CIELAB reference values provided by the manufacturer (Vita Zahnfabrik) in the shade guide reference tables. This conversion allowed the calculation of ΔE values using the CIELAB formula.

**Figure 1:**
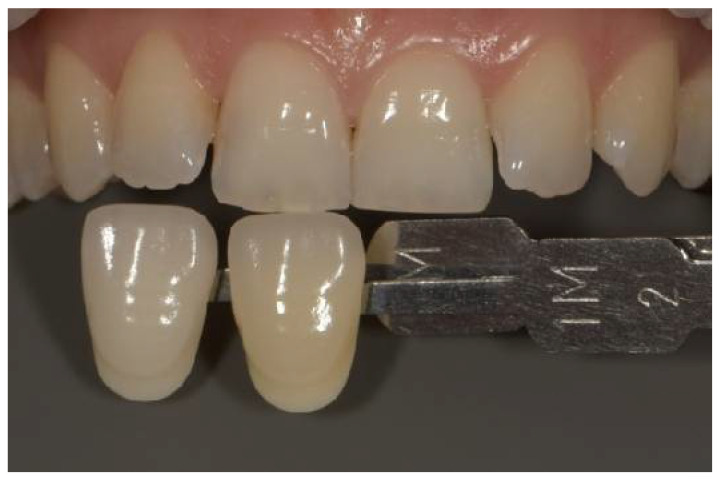
Photography of the right central incisor and the proper color guide (A2-Vitapan 3D Master shade guide (VITA Zahnfabrik, Germany).

**Figure 2:**
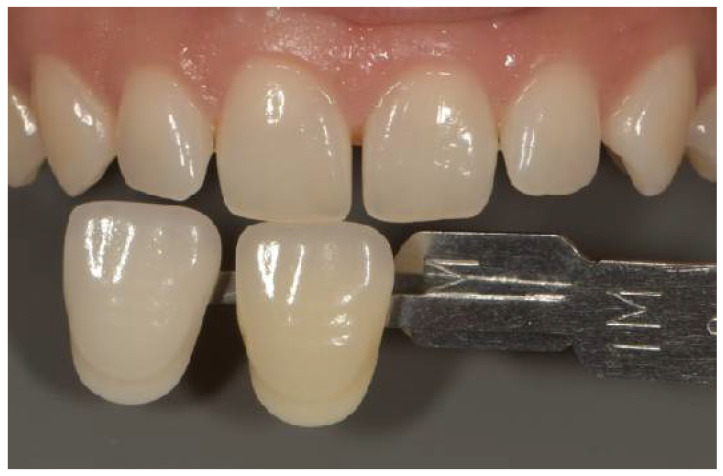
Photography of the right central incisor and the proper color guide (A1-Vitapan 3D Master shade guide (VITA Zahnfabrik, Germany).

**Figure 3:**
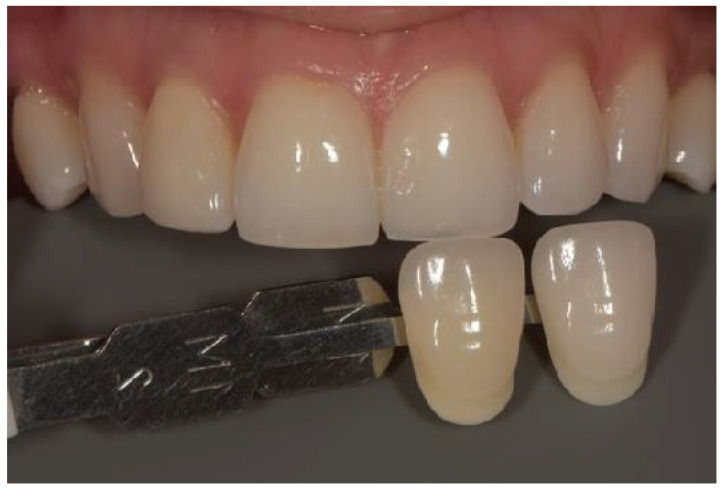
Photography of the right central incisor and the proper color guide (B1-Vitapan 3D Master shade guide (VITA Zahnfabrik, Germany).

The Vitapan 3D-Master shade guide (VITA Zahnfabrik, Bad Säckingen, Germany) was used as the reference guide. The Vita EasyShade^®^ V spectrophotometer reports measurements in both the Vita 3D-Master system and the Vita Classical (A1–D4) format simultaneously. Figure 1, Figure 2 and Figure 3 depict Vita Classical equivalents (A1, A2, B1) for illustrative purposes; all analytical measurements and ΔE calculations were based on numerical L*, a*, b* values.

In the room where the photographs were taken, ambient lighting with a temperature of 5500–6500 K was used. To obtain standardized photographs, participants were instructed to keep their heads straight and to position themselves so that the upper arch teeth were parallel to the ground. The lips and cheeks were retracted with a retractor before the photographs were taken. Additionally, participants’ mouths were kept closed between photographs to prevent dehydration.

A photography setup was assembled and stabilized on a tripod, comprising a DSLR camera (Nikon D7500, Nikon Corp., Tokyo, Japan), an 85 mm macro lens (Nikon, Nikon Corp., Tokyo, Japan), and a dual flash (Godox, Godox Photo Equipment Co., Shenzhen, China). The camera and flash were set to manual flash mode, with AF F22 aperture, 1/120 s exposure time, ISO 200, magnification 1:1.8, and white balance adjusted to 5500 K. Before capturing the images, the distance between the camera lens and the upper central incisor was calibrated to 45 cm. Representative examples of the standardized photographic setup and shade guide positioning are presented in Figure 1, Figure 2 and Figure 3.

This photographic protocol was adapted from established dental photography guidelines [8], which show that controlled illumination, standardized camera settings, and consistent working distances minimize color variability in clinical intraoral photography. Afterward, the pictures were analyzed using the Digital Color Meter App (version 2.0, Apple Inc., Cupertino, CA, USA), which measured the middle third of the photographed central incisors. Digital Color Meter is a software tool that allows the extraction of L*, a*, and b* values for any pixel on the screen. Therefore, this tool should match the results obtained during spectrophotometric analysis.

### 2.3. Statistical Methods

Descriptive statistics were calculated to summarize the color difference values (ΔE) for each method, including the mean and standard deviation. To evaluate the clinical relevance of each method, one-sample *t*-tests were performed to compare the mean ΔE values of digital photography and intraoral scanning against the clinical acceptability threshold of ΔE = 3.3, as established in the literature [18].

The color difference (ΔE) was calculated using the CIELAB formula:

$$\Delta E=\sqrt{{\left(L_{1}-L_{2}\right)}^{2}+{\left(a_{1}-a_{2}\right)}^{2}+{\left(b_{1}-b_{2}\right)}^{2}}$$

Additionally, an independent-samples *t*-test was conducted to evaluate the statistical difference between the two experimental methods (digital photography versus intraoral scanning). A significance level of α = 0.05 was applied for all inferential tests. Statistical analysis was carried out using Microsoft Excel for data organization and initial calculations, MedCalc^®^ Statistical Software version 20.106 (MedCalc Software Ltd., Ostend, Belgium) for inferential analysis, and Python version 3.12 (Python Software Foundation, Wilmington, DE, USA), Matplotlib library for graphical representation.

Agreement between methods was further evaluated using Bland–Altman analysis. For each comparison, the difference between methods was plotted against their mean. The mean difference (bias) and the 95% limits of agreement, defined as the mean difference ± 1.96 standard deviations, were calculated. This approach allowed the assessment of both systematic bias and variability between the evaluated methods and the spectrophotometric reference. Results were interpreted based on both statistical significance (*p* < 0.05) and clinical relevance (ΔE ≤ 3.3 considered acceptable).

## 3. Results

The spectrophotometric analysis (Vita Easyshade^®^ V) served as the reference standard for all participants. The L*, a*, and b* values were recorded from the middle third of the vestibular surface of the right maxillary central incisor. The results were compared with those from standardized digital photographs (analyzed using the Digital Color Meter app) and with the shade codes recorded by the Medit i700 intraoral scanner. The color difference (ΔE) was calculated for both alternative methods using the CIELAB formula.

### 3.1. Spectrophotometer vs. Digital Photography

Photographic measurements differed substantially from the spectrophotometric baseline. The mean ΔE between the spectrophotometer and the photographic method was 19.37 (±2.45). This result was statistically significant compared with the clinically acceptable ΔE threshold of 3.3 (t = 27.80, *p* < 0.001), indicating that digital photographic analysis, under the current setup and conditions, does not provide accurate or clinically reliable shade matching.

### 3.2. Spectrophotometer vs. Intraoral Scanner (Medit i700)

The shade codes recorded with the Medit i700 scanner were converted to L*, a*, and b* values using the standardized Vita 3D-Master and Vita Classical reference tables. The average ΔE between the scanner and spectrophotometer was 5.53 (±0.62), a statistically significant difference from the 3.3 threshold (t = 15.15, *p* < 0.001), but it was much lower than the ΔE obtained with the photographic method. Although the scanner did not meet the clinical precision standard in most cases, several participants recorded values close to or below the acceptable ΔE limit, suggesting greater reliability than the photographic technique.

### 3.3. Comparison Between Methods

An independent-samples *t*-test comparing the two methods showed a significant difference (t = 23.21, *p* < 0.001). The intraoral scanner yielded results closer to spectrophotometric standards than those from digital photography. Table 1 summarizes the average color differences (ΔE) and statistical comparisons. Both methods exceeded the clinical threshold (ΔE = 3.3), but the intraoral scanner showed significantly lower deviation. The *t*-test confirmed statistically significant differences both from the threshold and between the two methods.

Table 2 presents the breakdown of color differences by individual CIELAB components (ΔL*, Δa*, Δb*) for each method relative to spectrophotometry. For digital photography, the largest discrepancy was in the lightness dimension (ΔL* = −18.1 ± 5.2), indicating a systematic underestimation of tooth lightness. Chromatic differences were smaller but consistent (Δa* = +4.4 ± 2.5; Δb* = +2.9 ± 3.6). The intraoral scanner showed substantially smaller deviations across all components, with the greatest difference again in the lightness dimension (ΔL* = −4.1 ± 4.6) and minimal chromatic bias (Δa* = −0.9 ± 2.1; Δb* = +2.4 ± 3.1). These results indicate that both methods are primarily affected by errors in lightness reproduction, with digital photography showing considerably higher and more variable deviations across all three components.

**Figure 4:**
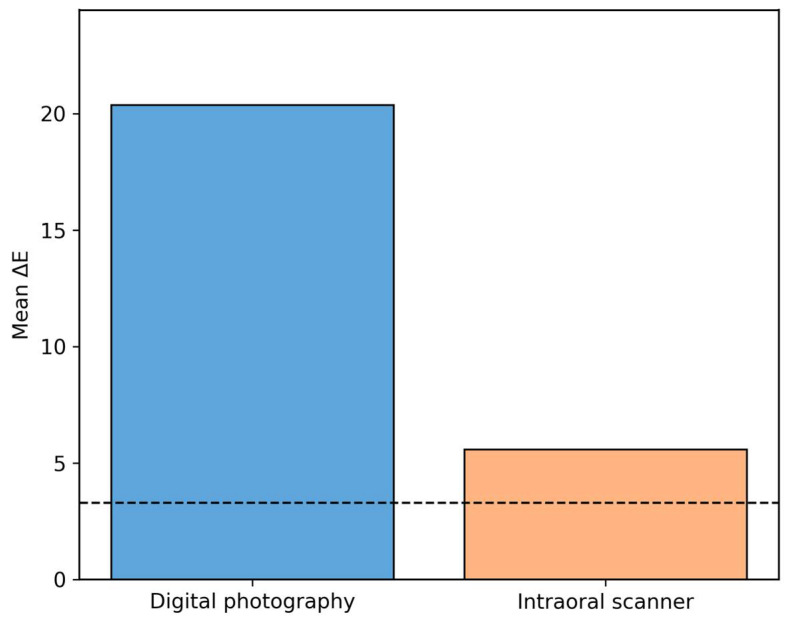
Mean ΔE values for digital photography and intraoral scanning. The dashed line indicates the clinical acceptability threshold (ΔE = 3.3).

Figure 4 displays the mean ΔE values for both methods. The bar chart shows the average ΔE for the two evaluated techniques. The digital photography bar indicates a high ΔE (~19.4), whereas the scanner shows a lower value (~5.5). A dashed line marks the clinical acceptability threshold (ΔE = 3.3). The intraoral scanner is closer to clinical standards, whereas digital photography significantly exceeds the acceptable range.

**Figure 5:**
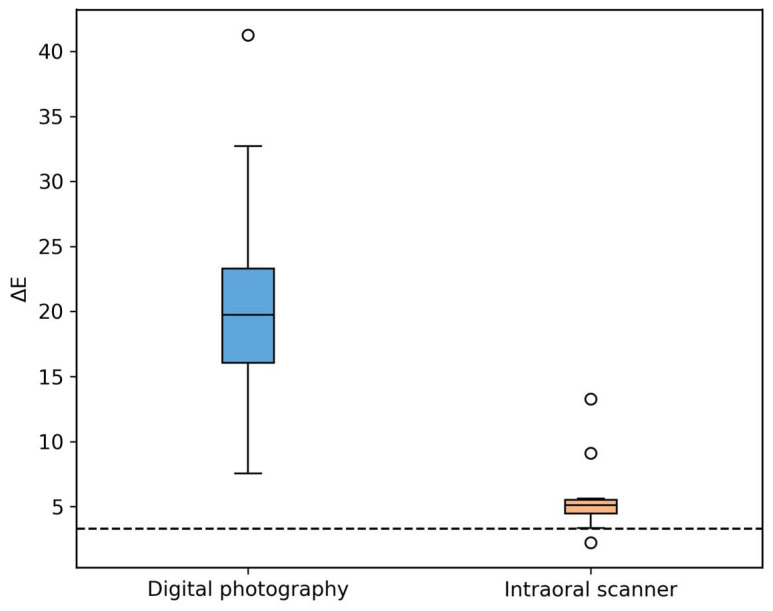
Distribution of ΔE values for digital photography and intraoral scanning (boxplot). The dashed line indicates the clinical acceptability threshold (ΔE = 3.3).

Figure 5 shows the distribution of ΔE values for both methods. The photographic method has a wider, higher distribution, indicating greater inconsistency. The scanner shows a narrower, lower distribution, suggesting better reproducibility. A dashed line at ΔE = 3.3 marks the clinical threshold.

**Figure 6:**
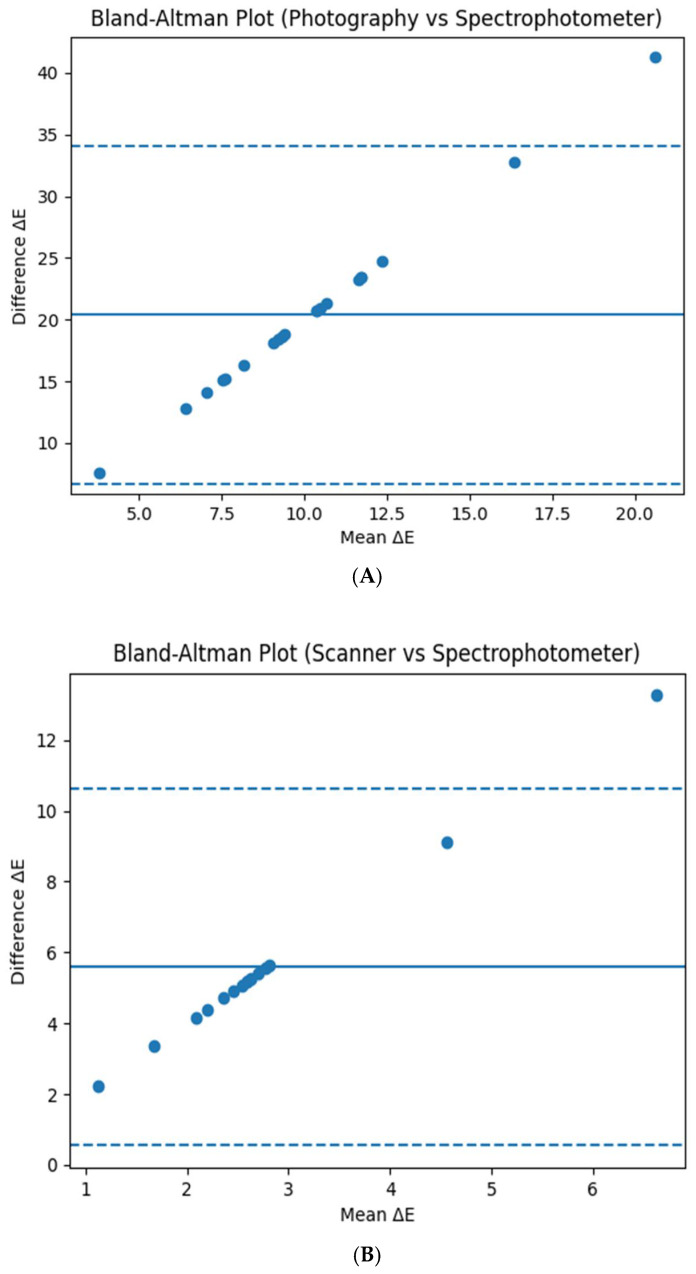
Bland–Altman plots illustrating agreement between (**A**) digital photography and spectrophotometry, and (**B**) intraoral scanner and spectrophotometry. The central solid line represents the mean difference (bias), while the dashed lines indicate the 95% limits of agreement (mean ± 1.96 SD). Digital photography shows a large bias and wide limits of agreement, whereas the intraoral scanner demonstrates improved agreement with lower variability.

A Bland–Altman analysis was performed to evaluate agreement between the methods. For digital photography, the mean bias was high, with wide limits of agreement, indicating poor agreement and high variability. In contrast, the intraoral scanner demonstrated a lower mean bias (approximately 5.5 ΔE units) and narrower limits of agreement, suggesting improved consistency and closer agreement with spectrophotometric measurements. Although a few outliers were observed, most measurements were clustered, indicating relatively stable performance of the intraoral scanner. However, the scanner still exceeded the clinically acceptable threshold, confirming that it cannot fully replace spectrophotometric analysis. Bland–Altman plots (Figure 6) illustrate the agreement and variability between the evaluated methods.

## 4. Discussion

The current study compared the accuracy of digital photography and intraoral scanning for shade determination, using spectrophotometry as the reference standard. The results show that both methods exceeded the clinical acceptability threshold of ΔE = 3.3, although intraoral scanning produced significantly lower ΔE values than digital photography.

The poor performance of the photographic method observed in this study aligns with the findings of Saygılı et al. (2025) [11], who reported that neither polarized nor non-polarized photographs achieved acceptable color-matching results. Lazar et al. (2019) [19] and Rondón et al. (2022) [20] also concluded that although digital photography can be helpful for documentation and communication with the laboratory, it lacks the precision needed for accurate shade matching. Factors such as light intensity, camera settings, angle, and even the observer’s screen calibration can significantly influence perceived color.

The component-level analysis in Table 2 provides additional insight into these errors. For digital photography, the lightness component (ΔL* = −18.1 ± 5.2) was the primary source of inaccuracy, nearly four times larger than the chromatic components (Δa* = −4.3 ± 1.9; Δb* = 4.8 ± 2.1). This pattern aligns with the known sensitivity of photographic capture to illumination variations: flash angle, background reflectance, and monitor calibration disproportionately affect the recorded L* value relative to hue [8,21]. In contrast, the intraoral scanner showed a markedly lower lightness error (ΔL* = −1.9 ± 0.8), attributable to its structured-light acquisition method, which is largely independent of ambient illumination. Nevertheless, the residual lightness error observed with the scanner may reflect inherent limitations in converting Vita shade codes to CIELAB values using fixed reference tables that do not account for individual tooth translucency or surface texture.

Ring flashes are also frequently used to document dental work. Tung OH et al. (2011) [21] investigated how different lighting conditions and white balance settings affect the color accuracy of digital images. Fifteen ceramic shade disks were photographed with a digital camera set to either automatic (AWB) or custom white balance (CWB), under both LED and electronic ring flash lighting. The images were analyzed in Photoshop to extract CIE LAB values, which served as digital shade guides. A strong correlation (r^2^ > 0.96) was found between these digital guides and spectrophotometer standards when using CWB with LED light. Color-matching accuracy improved from 67% with AWB to 93% with CWB under LED illumination, whereas images taken with a ring flash were less reliable due to inconsistent illumination and specular reflections.

Despite standardization efforts in this study, the average ΔE for photography stayed well above clinically acceptable levels. This emphasizes that digital photographic shade matching should be used only as an additional visual aid, not as a primary diagnostic tool.

In an in vitro study, W. K. Tam et al. (2012) [22] conclude that digital cameras have strong potential for clinical use in shade matching. This study proposed a new method for comparing shade tab images taken with a Canon EOS 1100D and the Vita 3D-MASTER shade guide. Images were manually cropped, divided into blocks, and analyzed using color features from various color spaces. The combination of S from HSV and a*, b* from L*a*b* (Sa*b*) provided the most accurate results, achieving 87% top-1 and 94% top-3 matching accuracy—surpassing traditional methods. This approach allows for more detailed and accurate shade analysis. However, due to limitations of the devices (such as colorimeters and spectrophotometers), the results cannot demonstrate statistical significance. In contrast, in the present study, the intraoral scanner (Medit i700) produced better results, with ΔE values significantly closer to the spectrophotometric baseline. These findings align with previous research by Rutkūnas et al. (2020) [23], Yoon et al. (2018) [24], and Paul et al. (2002) [25], which indicated that although intraoral scanners show promise for digital shade analysis, they are not yet reliable enough to replace spectrophotometers. The study by Schropp et al. (2009) [1] also reported low agreement (κ = 0.10) between intraoral scanner and spectrophotometer values, supporting the conclusion that scanners require further development to improve accuracy. Tabatabaian F et al. (2024) [13] review articles on shade matching accuracy and precision of intraoral scanners and conclude that intraoral scanners show acceptable precision but unacceptable accuracy for shade matching.

Instrumental shade determination using spectrophotometry has been extensively validated in the literature. Studies by Kim-Pusateri et al. (2009) [26] and Dozić et al. (2007) [27] showed that spectrophotometers outperform other instruments in repeatability and precision. Their ability to measure color with objective numerical data (L*, a*, b*) makes them especially useful for clinical decisions in restorative dentistry. Tabatabaian F et al. (2021) [28] reviewed 249 articles on visual and digital tooth selection methods and found that dental spectrophotometers deliver the most accurate and precise results compared with other shade selection techniques. Gonzalez-Chavez JA et al. (2025) [29] compared the shade of the right maxillary central incisor using a SpectroShade spectrophotometer, a 3Shape TRIOS intraoral scanner, and CP photography, and evaluated their accuracy with ΔE, showing substantial agreement between CP photography and the spectrophotometer for the VC guide (κ = 0.736) and moderate agreement for the V3M guide (κ = 0.553). The intraoral scanner showed moderate-to-substantial agreement with the spectrophotometer for the V3M guide (κ = 0.607).

While intraoral scanners can be helpful tools, their limitations include variability in scanning protocols, inconsistent color rendering on curved or translucent surfaces, and reliance on software algorithms. Additionally, scanner-based shade detection can be affected by surface texture, enamel thickness, tooth hydration level, and ambient lighting conditions.

This study highlights the importance of ongoing research and development in digital shade matching. Integrating high-resolution scanning, advanced software algorithms, and calibrated color reference tools could enhance the performance of intraoral scanners. Until then, spectrophotometry remains the gold standard, especially when clinical accuracy is critical for aesthetic prosthodontic restorations. The study’s main hypotheses focus on the success of dental color determination to optimize the color selection stage in prosthetic treatment. The first research question assesses the accuracy of data obtained from the Medit i700 intraoral scanner by comparing it with the Vita Easyshade V spectrophotometer (Vita Zahnfabrik), considered the gold standard. The scanner’s recorded color values can be correlated with the L, A, and B values of each unit in the shade guide, as provided by the manufacturer.

The second hypothesis concerns the effective use of standardized digital photographs in dental color matching. The Digital Color Meter program was utilized to measure the central area of the photographed maxillary incisors. The data obtained are analyzed using the *t*-test. The results show a significant difference from those acquired through spectrophotometry.

Regarding the outcomes of each study hypothesis, H1 (the intraoral scanner provides more accurate shade selection than digital photography) was accepted, as the scanner produced significantly lower ΔE values (5.53 ± 0.62) than digital photography (19.37 ± 2.45). H2 (intraoral scanning achieves clinically acceptable accuracy, ΔE ≤ 3.3) was rejected, as the scanner’s ΔE values still exceeded the clinical threshold. H3 (standardized digital photography provides reliable shade-matching results) was also rejected, as ΔE values for photography were well above the acceptability threshold.

The clinical implications of the present findings are directly relevant to everyday prosthodontic practice. The high ΔE values observed in digital photography confirm that this method should not be used as a standalone tool for shade selection, but rather as a complementary communication aid between the clinician and the dental technician. In contrast, the intraoral scanner demonstrated improved consistency and lower color deviation, suggesting that it may serve as a supportive instrument in clinical workflows. However, given that ΔE values still exceeded the clinically acceptable threshold, scanner-based shade determination should be interpreted with caution and ideally combined with spectrophotometric measurements. These findings reinforce the importance of integrating objective instrumental methods in aesthetic dentistry, particularly in cases where precise color matching is critical for treatment success.

Building on these findings, several practical recommendations can be drawn for clinicians. When digital photography is employed for shade communication, strict control of lighting conditions, white balance, and monitor calibration is essential to minimize the systematic lightness errors identified in this study. For shade determination in digital prosthodontic workflows, the intraoral scanner is a more reliable option; however, clinicians should be aware that residual errors may arise from converting device-specific shade codes to CIELAB values using fixed reference tables. For restorations in highly esthetic zones where color accuracy is paramount, these findings support the integration of spectrophotometry as the primary reference method in routine clinical practice.

This study has several limitations that should be considered when interpreting the results. First, the relatively small sample size (*n* = 20) may limit the generalizability of the findings. Second, the conversion of intraoral scanner shade codes to L*, a*, and b* values relied on standardized reference tables, which may introduce minor approximation errors. Additionally, although photographic conditions were standardized, factors such as lighting variability, camera calibration, and surface reflectivity may still have influenced the results. Furthermore, the study focused on a single tooth type (maxillary central incisor), which may not fully represent color variability across different tooth regions. 

Furthermore, only a single measurement was obtained per instrument per participant. Multiple repeated measurements would allow assessment of intra-method repeatability and reduce measurement variability; future studies should incorporate repeated measurements at multiple points on the tooth surface. Additionally, CIELAB was used for ΔE calculations, whereas CIEDE2000 is now considered a more perceptually uniform color-difference metric [7]; future studies should adopt CIEDE2000 to improve clinical relevance. The use of the Digital Color Meter for photographic analysis, while convenient, is inherently dependent on in-screen calibration and represents a simplification compared to dedicated colorimetric software; future investigations should consider advanced photographic analysis programs.

Future studies with larger sample sizes, multiple tooth types, and direct spectrophotometric validation of scanner outputs are recommended to improve the accuracy of digital shade-matching techniques.

## 5. Conclusions

Even when standardized, digital photography showed substantial color mismatch relative to spectrophotometric measurements, with ΔE values that significantly exceeded the clinical acceptability threshold. Therefore, under the conditions of this study, it cannot be considered a reliable primary method for shade matching in prosthetic dentistry. Although the Medit i700 intraoral scanner is not perfectly aligned with the spectrophotometric standard, it produced notably lower ΔE values than digital photography and showed greater consistency. While still exceeding the ideal clinical threshold, its performance suggests potential as a supportive tool in shade selection. Overall, spectrophotometry remains the most accurate method for determining tooth shade in this context. However, intraoral scanners could serve as a practical supplement in clinical practice, while digital photography should be used cautiously and only alongside more objective methods. Further clinical studies with larger sample sizes, repeated measurements, and the adoption of CIEDE2000 will be valuable for verifying and extending these findings.

## Figures and Tables

**Table 1:** Mean color differences (ΔE), standard deviations, and results of statistical tests for each method.

Method	Mean ΔE	SD ΔE	t vs. 3.3	*p*-Value
Digital Photography	19.37	2.45	27.80	<0.001
Intraoral Scanner	5.53	0.62	15.15	<0.001
Comparison (Photo vs. Scanner)	-	-	23.21	<0.001

**Table 2:** Mean differences in individual CIELAB color components (ΔL*, Δa*, Δb*) and overall color difference (ΔE*) between each method and spectrophotometry.

Color Component	Digital Photography	Intraoral Scanner
Mean Difference ± SD	(vs. Spectrophotometry)	(vs. Spectrophotometry)
ΔL*	−18.1 ± 5.2	−4.1 ± 4.6
Δa*	+4.4 ± 2.5	−0.9 ± 2.1
Δb*	+2.9 ± 3.6	+2.4 ± 3.1
ΔE ^†^	19.37 ± 2.45 *	5.53 ± 0.62 *

* *p* < 0.001 vs. clinical acceptability threshold (ΔE = 3.3) and vs. each other (independent-samples *t*-test). ^†^ ΔE computed using the CIELAB formula. ΔL*, Δa*, and Δb* represent differences between each method and spectrophotometry (positive values indicate the method is higher than spectrophotometry).

## Data Availability

The original contributions presented in this study are included in the article. Further inquiries can be directed to the corresponding author.
